# Adenomatoid tumor of the testis mimicking malignant testicular cancer on multiparametric ultrasound

**DOI:** 10.1186/s40001-018-0301-5

**Published:** 2018-01-11

**Authors:** Renate Pichler, Gennadi Tulchiner, Fabian Steinkohl, Afschin Soleiman, Wolfgang Horninger, Isabel Maria Heidegger, Friedrich Aigner

**Affiliations:** 1 Department of Urology, Medical University of Innsbruck, Anichstreet 35, 6020 Innsbruck, Austria; 2 Department of Radiology, Medical University of Innsbruck, Innsbruck, Austria; 3 Clinical Pathology and Cytodiagnostics, Tyrolean State Hospitals Ltd., Innsbruck, Austria

**Keywords:** Adenomatoid tumor, Testis, Tunica albuginea, Ultrasound, Elastography

## Abstract

**Background:**

Adenomatoid tumor is one of the most common histological subtypes of paratesticular cancer arising from the epididymis. In very rare cases, these tumors appear as intratesticular lesions originating in the tunica albuginea, representing a diagnostic challenge.

**Case presentation:**

We present a case of a 51-year-old man with a small (0.9 cm) hyperechoic lesion of the left testicle mimicking testicular cancer on multiparametric ultrasound. The lesion was localized in the peripheral zone, confirming vascularization and increased stiffness on contrast-enhanced ultrasound and real-time elastography. Preoperative tumor markers and hormone levels were within normal ranges. Staging computed tomography was negative. Organ-sparing surgery with tumor enucleation and frozen section analysis was performed, confirming testicular adenomatoid tumor.

**Conclusion:**

Currently, no typical ultrasound features can definitively distinguish intratesticular adenomatoid tumors from malignant testicular masses. Thus, a surgical approach is almost always considered in such a case for both diagnostic and therapeutic purposes.

## Background

Adenomatoid tumors in the male urogenital tract are benign mesenchymal tumors mostly located in the lower or upper pole of the epididymis [1], being responsible for more than 30% of all paratesticular cancers [2, 3]. In contrast, adenomatoid tumors are observed markedly less as intratesticular lesions. In this case, the tumor originates in the tunica albuginea being responsible for their peripheral location [4]. However, the majority of cases appear nonspecific and variable [5], without typical imaging features [3, 4]. Unfortunately, no ultrasound features can definitively distinguish an adenomatoid tumor from malignant testicular masses [6, 7], although real-time elastography (RTE) [8, 9] and contrast-enhanced ultrasound (CEUS) [10] showed promising results with increased diagnostic accuracy in differentiating malignant from benign testicular lesions. Currently, a surgical approach is almost always considered in such a case for both diagnostic and therapeutic purposes.

We present the case of a patient with adenomatoid tumor of the left testicle mimicking imaging characteristics of a malignant testicular cancer on multiparametric ultrasound.

## Case report

A 51-year-old man was referred at the urological department due to the primary suspicion of a left testicular cancer. Staging computed tomography of the thorax, abdomen, and pelvis was negative for lymphatic or hematogeneous metastatic tumor spread. Moreover, testicular tumor markers such as α-fetoprotein (AFP) and beta-human chorionic gonadotropin (β-hCG), and preoperative hormone levels (testosterone, LH, FSH) were within normal ranges.

**Fig. 1:**
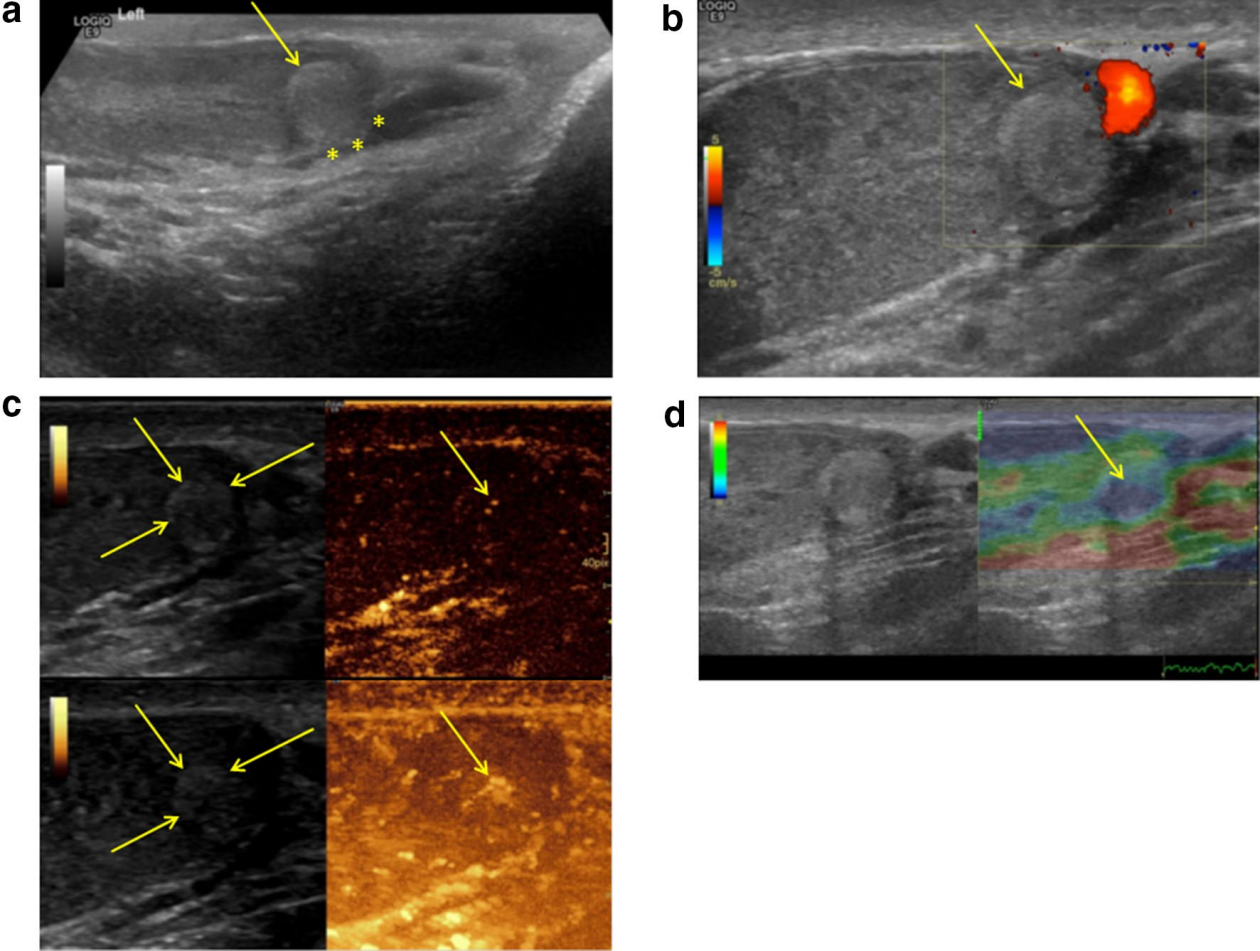
**a** Peripheral, rounded, and hyperechoic lower pole lesion of the left testicle (arrow), connected with the tunica albuginea (asterisk) on B-mode sonography. **b** No vascularization on baseline color and power Doppler sonography (arrow). **c** In contrast to color Doppler sonography, the lesion was contrast-absorbing indicating vascularity on CEUS. **d** RTE demonstrated the lesion with increased stiffness (blue, arrow)

Testicular ultrasound confirmed a 0.9 × 0.9 × 1.0 cm rounded, peripheral, and hyperechoic lesion of the lower pole of the left testicle (Fig. 1a) connected with the tunica albuginea, without pathological signs of the right testicle. No exact vascular signs or vascular flow within this lesion were reproducible on baseline color and power Doppler sonography (Fig. 1b). Contrast-enhanced ultrasound (LOGIQ E9 ultrasound machine by GE Healthcare, Fairfield, Connecticut, USA) was performed as an additional diagnostic tool to clarify the vascularization of the suspicious lesion. Interestingly, the lesion was contrast-absorbing after bolus contrast agent application of 2.4 cc (SonoVue, Bracco, Milan, Italy), Fig. 1c. Moreover, real-time elastography (RTE) of the left testis was performed to evaluate the tissue elasticity of the tumor mass, showing a hard lesion (Fig. 1d). In summary, multiparametric ultrasound confirmed a vascularized and hardened lesion, therefore being suspicious of testicular cancer.

Next, inguinal exploration with organ-sparing surgery and tumor enucleation was performed without ischemia. Intraoperative frozen section analysis of the tumor and tumor bed biopsies was performed, providing no evidence of malignant germ cell tumor or testicular intraepithelial neoplasia (TIN). Final pathology report confirmed a completely removed adenomatoid tumor of the left testicle arising from the tunica albuginea.

## Discussion

Adenomatoid tumors that arise very rarely in the testicles in contrast to the epididymis are a difficult diagnostic challenge in the clinical practice that may lead to unnecessary orchiectomy [11]. These tumors are presented typically as small, well-demarcated, and peripheral lesions, most often localized to the lower pole of the testis, and usually occurring between the third and fifth decades of life [11]. Nonspecific sonomorphological features of adenomatoid tumors of the testis make diagnosis even more difficult, showing no reliable echoic pattern [11–13]. Thus, echogenicity of intratesticular adenomatoid tumors can range from isoechoic, hypoechoic to hyperechoic [11]. Novel imaging techniques such as RTE and CEUS are currently introduced as additional diagnostic tools to increase the sensitivity and specificity in differentiating dignity of testicular lesions before surgery [8, 9]. For example, Auer et al. [14] evaluated the diagnostic accuracy of multiparametric ultrasonography (US) consisting of gray-scale US, color Doppler US, strain elastography, and contrast agent-enhanced US in the assessment of intratesticular lesions. Sensitivities and specificities were 66.7 and 88.4% for color Doppler US, 100 and 76.7% for contrast-enhanced US, and 100 and 72.1% for strain elastography, respectively. However, in our case, multiparametric ultrasound mimicked testicular cancer, confirming a vascularized and hard lesion of 0.9 cm. Nevertheless, fine needle aspiration cytology (FNAC) seems to be an attractive diagnostic method in case when adenomatoid tumor is supposed [15, 16]. Cytological features include dilated tubular structures lined by epithelial cells with round or oval nuclei, with clear and vacuolated cytoplasm in Papanicolaou staining, staining pink with Giemsa stain [15, 16]. Nevertheless, we did not perform FNAC for further diagnostic evaluation as RTE and CEUS confirmed all sonographic criteria of a malignant testicular cancer. Concerning the therapeutic management, intratesticular adenomatoid tumors are treated surgically with a curative approach, favoring an “organ-sparing” strategy whenever possible [17]. According to the European Association of Urology (EAU) guidelines, testicular “organ-sparing surgery” is possible in special cases, when a patient presents with a tumor in a solitary testis, bilateral testicular cancers, or metachronous contralateral tumors. However, negative preoperative tumor markers and normal hormone levels with a tumor volume < 30% of the testicular volume represent an essential condition for this organ-preserving surgical approach [18]. In our patient, we performed inguinal tumor enucleation without ischemia and frozen section analysis despite typical features of a malignant testicular cancer on RTE and CEUS, as the lesion was very small with 0.9 cm and located peripheral, with normal testosterone levels and negative imaging and testicular tumor markers preoperatively.

## Conclusion

In conclusion, no typical ultrasound features can definitively distinguish intratesticular adenomatoid tumors from malignant testicular masses. Thus, a surgical approach is almost always considered in such a case for both diagnostic and therapeutic purposes.

## Abbreviations

CEUS: contrast-enhanced ultrasound
EAU: European Association of Urology
FNAC: fine needle aspiration cytology
FSH: follicle-stimulating hormone
LH: luteinizing hormone
RTE: real-time elastography
TIN: testicular intraepithelial neoplasia
